# Acupoint for angina pectoris: A protocol for systematic review and meta-analysis

**DOI:** 10.1097/MD.0000000000024080

**Published:** 2021-01-22

**Authors:** Li Jiang, Zhaoxia Deng, Hongcai Zhang, Yuan Li, Tingting Wang, Wen Xie

**Affiliations:** a Hospital of Chengdu University of Traditional Chinese Medicine, Chengdu, Sichuan Province; b Chengdu University of Traditional Chinese Medicine, PR China.

**Keywords:** acupoint therapies, angina pectoris, protocol, systematic review

## Abstract

**Introduction:**

Patients with angina pectoris (AP) often experience heavy psychological distress, especially anxiety and depression, which results in poorer quality of life, shorter survival time. Acupoint therapies, including massage, acupuncture, acupoints injection, acupressure, and moxibustion, showed clinical and long-lasting benefits for AP, but the efficiency of acupoint therapies was poorly evaluated. The current review is attempted to evaluate the efficacy and safety of the different acupoint-based therapies for AP.

**Methods and analysis:**

A literature search will be conducted in MEDLINE, EMBASE, Web of Science, Cochrane Library, Web of Science, PubMed, Science Direct, Wan Fang Data Knowledge Service Platform, Chinese Biomedical Literature Database (CBM), Chinese Scientific Journal Database (VIP database), and China National Knowledge Infrastructure (CNKI). Observational studies regarding the association between liver cancer and depression and anxiety written in English or Chinese will be included. Study inclusion, data extraction, and quality assessment will be performed independently by 2 reviewers. We will use RevMan V.5.0 and STATA V.12.0 software for statistical analysis. The *I*^2^ test will be used to identify the extent of heterogeneity. Publication bias will be assessed by generating a funnel plot and performing the Begg and Egger test. The quality of the systematic review will be evaluated using the Measurement Tool to Assess Systematic Reviews (AMSTAR) and Grading of Recommendations Assessment Development and Evaluation (GRADE) criteria. With the permitted numeric data, we will carry out a meta-analysis.

**Results:**

This study will provide a high-quality synthesis of pain VAS and functional disability or the quality of life, the success treatment rate, the recurrent rate and the complications rate to assess the effectiveness and safety of acupoint for AP patients. This systematic review will provide evidence to judge whether acupoint is an effective intervention for patients with AP.

**Conclusion:**

This systematic review and meta-analysis will provide evidence to judge whether acupoint is an effective intervention for patients with AP and provide evidence for designing early targeted interventions for high-risk survivors that can attenuate negative reactions.

**PROSPERO registration number:**

10.17605/OSF.IO/VNXWE.

## 1 Introduction

Cardiovascular diseases are currently the leading cause of death in the world. According to the 2017 Global Burden of Disease study, 31.80% of the deaths (17.79 million people) were caused by cardiovascular diseases, and 4.38 million deaths (41.89%) in China were attributed to this. In the United States, 72% of the patients who died from cardiovascular disease are over 65, mainly CHD.^[1]^ China now has 330 million cases of cardiovascular diseases, including 11 million cases of CHD. AP of CHD is a kind of clinical symptom caused by coronary artery atherosclerosis which leads to insufficient blood supply. Caused by transient myocardial ischemia and hypoxia, angina pectoris (AP) is very common in the clinical diagnosis and treatment of cardiovascular medicine, manifesting as paroxysmal retrosternal pain.^[2]^ AP is also a sign that someone is at increased risk of heart disease, cardiac arrest, and sudden cardiac death. AP affects more than 560,000 people in the United States each year. According to data from the European Society of Cardiology, the prevalence of AP in most European countries is estimated at 20,000 to 40,000 per million people.^[3]^ At present, the drug treatments for AP mainly include beta blockers, nitric acid ester, calcium channel blockers, antiplatelet drugs and so on. Although, drug therapy alone can relieve some symptoms, it can cause adverse reactions, which may lead to serious clinical consequences and affect patients’ quality of life. In addition to conventional drugs, the European Society of Cardiology also recommends nondrug treatments, including risk factors elimination, lifestyle changes, and vascular reconstruction treatments.^[4]^ In real-world clinical practice, patients are eager to seek safer and more effective complementary and alternative therapies to relieve angina symptoms.

Acupuncture, as the most famous complementary and alternative medicine method, has been used to prevent and treat many diseases for more than 2000 years. According to the basic theories of traditional Chinese medicine, the mechanism of acupuncture therapy is to stimulate the body's specific meridian points, stimulate the meridian qi and blood, dredge the meridian, and reconcile yin and yang, so as to achieve the purpose of strengthening the body and eliminating pathogenic factors. A large number of clinical reports have proved that acupuncture therapy can effectively treat a variety of cardiovascular diseases, such as CHD, hypertension, heart failure, arrhythmia and so on.^[5–8]^ Currently published studies show that acupuncture can improve the symptoms of patients with AP, and has a good effect on reducing the frequency of AP.^[9]^ However, the evidence of acupuncture treatment of AP is far from meeting the modern clinical norms. In order to provide reliable evidence that acupuncture can effectively treat AP, we will conduct a systematic review and meta-analysis of evidence-based medicine. The validated results will provide clinicians with treatment options to further improve the survival and quality of life of patients with AP.

## 2 Objective

The primary purpose of this study protocol is to evaluate the SAQ (Seattle Angina Questionnaiire) of acupoint therapies in AP patients and examine the efficacy of acupoint therapies in improving ADL and QoL in individuals with AP.

## 3 Methods

### 3.1 Inclusion criteria for study selection

#### 3.1.1 Types of studies

Observational studies with available data of the acupoint therapies for the management of **angina pectoris** will be included. Cross-sectional, cohort and case–control studies (with a sample of at least 30) will be included. Case reports, case series, opinion papers, qualitative research, letters to the editor, comments, conference proceedings, policy papers, reviews and meta-analyses, study protocols without baseline data, and animal studies will be excluded.

#### 3.1.2 Types of patients

Study populations inclusion criteria will be all patients diagnosed with **angina pectoris** and there are no restrictions on population ages, race, and education status.

#### 3.1.3 Types of outcome measures

1. Improvement in patients with AP as measured by the SAQ
2. The secondary outcomes are reduction in other scales or questionnaires evaluating pain or functional disability or the quality of life; the success treatment rate (after treatment the participants with a reduction of scales = 50% comparing to baseline), the recurrent rate and the complications rate.

### 3.2 Search methods for the identification of studies

#### 3.2.1 Data sources

The following databases will be searched: Web of Science, PubMed, Science Direct, Wan Fang Data Knowledge Service Platform, Chinese Biomedical Literature Database (CBM), Chinese Scientific Journal Database (VIP database), China National Knowledge Infrastructure (CNKI), and EMBASE. We will also conduct unpublished academic research data. Databases will be searched from inception to December 2019. The reference lists of review articles will be conducted and the following search terms will be used: prostate cancer, breast carcinoma, breast tumor, mammary cancer, mammary adenocarcinoma, risk factors, anxiety, anxious, depression, depressive disorder, cross-sectional study, cohort study, case-control study. And we will take the search strategy which is obtainable in Supplemental Digital Content (Appendix A) for searching the database. The authors will also search relevant trials from Clinical Trials.gov, Google Scholar and WHO International Clinical Trials Registry Platform.

### 3.3 Study selection

Studies imported into Endnote X8 software after removing duplicates will be independently reviewed by 2 authors based on the exclusion and inclusion criteria. The researchers will read the full text of relevant articles to confirm the final inclusion of studies. For unclear dates, the researchers will contact the author for details to determine whether this literature would be included. Any disagreement between reviewers will be resolved by discussion or a third rater.

### 3.4 Risk of bias assessment

The risk of bias/method quality of the included studies will be assessed independently by 2 authors at the study and outcome levels. Any disagreements will be solved by discussion or with arbitrament of the third author. In this study, we will use the Newcastle-Ottawa Scale to evaluate the quality of studies. This scale is a nonrandomized controlled trial quality evaluation instrument with scores ranging from 0 to 9; scores of 0–4 and 5–9 mean low quality and high quality, respectively.

### 3.5 Statistical collection and analysis

#### 3.5.1 Data extraction and management

The information that will be extracted include the first author's name, date of publication, journal, type of study (cross-sectional/cohort/case– control), country and region, sample size (N and male/ female), duration of the study, baseline age, diagnostic criteria for prostate cancer and anxiety and depression, incidence of anxiety and depression/mean and SD for anxiety and depression score, variables, OR values, 95% CI, and other relevant data for quality evaluation and risk of bias assessment. And the reasons for exclusion of studies while extracting will also be recorded. Possible risk factors include but are not limited to gender, age, occupation, marital status, education level, social support, alcohol status, smoking status, pathological type, cancer clinical stage, disease course and therapy method. The variables extracted will be adjusted in the process as it is likely that more and more variables that need to be included will turn up. Data gathering will be done by 2 reviewers independently. And if they are inconsistent in the process, they will discuss the results. A third reviewer will be consulted to resolve the doubts. The researchers will contact corresponding authors for unclear details.

#### 3.5.2 Measurements of prevalence and risk factors

RevMan V.5.0 will be used to calculate the OR values and 95% CIs of the reported risk factors for anxiety and depression in prostate cancer. When the CI of the OR value is not equal to 1 and *P* < .05, we believe it is statistically significant. The prevalence estimates reported by the individual studies will be extracted or converted into percent prevalence, and their respective SEs will be calculated. For the anxiety and depression scores, standardized mean difference will be used for analysis. We will use the Freeman-Tukey double arcsine transformation to stabilize the variance of study-specific prevalence. The prevalence of each study will be recalculated to confirm numerators and denominators, and adjustments will be made if necessary.

#### 3.5.3 Assessment of heterogeneity

The *I*^2^ test will be used to identify the extent of heterogeneity. When the *I*^2^ value is less than 50%, the fixed-effects model will be used. If the *I*^2^ value is higher than 50%, the random-effects model will be used, because we think the results of each study vary markedly.

In this study, factors of high heterogeneity will be removed one by one to find the source of any observed heterogeneity. The causes of heterogeneity may include differences in study design, statistical methods, and participants.

#### 3.5.4 Data synthesis

We will use RevMan V.5.0 and STATA V.12.0 software for analysis. Meta-analysis will be performed when the heterogeneity is low or the source could be found, although heterogeneity is high. A systematic narrative synthesis will be conducted if it is impossible to complete any meta-analysis. If there is significant heterogeneity, we will take the subgroup analysis.

#### 3.5.5 Subgroup analysis

Subgroup analysis will be done when data are available. The groups may be designed by country or region, diagnostic criteria for anxiety and depression, bias score, time since diagnosis (long-term vs short-term survivors), severity/staging of prostate cancer and study design.

#### 3.5.6 Assessment of reporting biases

We will assess publication bias by generating a funnel plot and performing the Begg and Egger test (*P* < .05 indicates the existence of publication bias).

#### 3.5.7 Quality control of the systematic review and meta-analysis

The methodological quality of the systematic review will be evaluated using the Measurement Tool to Assess Systematic Reviews (AMSTAR). The Grading of Recommendations Assessment, Development and Evaluation (GRADE) will also be used to evaluate the strength of evidence produced by the systematic review.

## 4 Discussion

At present, the specific mechanism of acupuncture treatment of AP is still unclear, this study will review the current research and provide effective evidence for acupuncture treatment of AP. The results of the systematic review and meta-analysis will not only provide clinicians with great help, but also improve the health and quality of work life of patients with AP. In this review, there might be some limitations. Firstly, in this review, only studies published in English or Chinese will be considered might cause a potential risk of publication bias. Secondly, different measurements and tools might lead to inconsistent levels of outcomes.

## Author contributions

WX is the guarantor of the article. The manuscript was drafted by LJ and ZD. HZ and ZD developed the search strategy. LJ and HC will independently screen the potential studies and extract data. ZD and LJ will assess the risk of bias and finish data synthesis. WX will arbitrate any disagreement and ensure that no errors occur during the review. All review authors critically reviewed, revised, and approved the subsequent and final version of the protocol.

**Conceptualization:** Hongcai Zhang.

**Data curation:** Zhaoxia Deng.

**Formal analysis:** Zhaoxia Deng.

**Investigation:** Hongcai Zhang, Yuan Li.

**Methodology:** Zhaoxia Deng.

**Project administration:** Wen Xie.

**Resources:** Li Jiang, Hongcai Zhang.

**Software:** Tingting Wang.

**Supervision:** Li Jiang, Zhaoxia Deng, Yuan Li, Tingting Wang, Wen Xie.

**Writing – review & editing:** Li Jiang, Zhaoxia Deng, Hongcai Zhang, Yuan Li, Tingting Wang.
